# Psychometric properties and measurement invariance across gender of the Chinese version of the Smartphone Application-Based Addiction Scale (SABAS) among Chinese college students

**DOI:** 10.1371/journal.pone.0323215

**Published:** 2025-05-21

**Authors:** Tian-Jiao Song, Hao Zhao

**Affiliations:** 1 School of Education, Shandong Women’s University, Jinan, Shandong, China; 2 Faculty of Education, Languages and Psychology, SEGI University, Kuala Lumpur, Malaysia; University of Sheffield, UNITED KINGDOM OF GREAT BRITAIN AND NORTHERN IRELAND

## Abstract

**Background:**

Smartphone addiction among college students is a common problem of concern, especially in China, and is associated with numerous psychological challenges. Nevertheless, a valid instrument to measure smartphone addiction in Chinese college students remains underdeveloped.

**Objective:**

To provide a valid research instrument for assessing smartphone addiction among Chinese college students, this study conducted a cross-cultural investigation by evaluating the psychometric properties of the Chinese Version of the Smartphone Application-Based Addiction Scale (SABAS) and its measurement invariance across gender among Chinese college students.

**Methods:**

The SABAS was translated into Chinese using the forward-backward method and the Chinese version of the SABAS (SABAS-CV) was completed by 644 Chinese college students. A random selection of 80 college students was made from the total sample, and they were assessed twice with a one-month interval. The reliability of the SABAS-CV was analyzed through internal consistency and test-retest reliability, while the validity was assessed via content validity, structural validity, and convergent validity. Additionally, this study tested the measurement invariance of the SABAS-CV across gender.

**Results:**

The SABAS-CV demonstrated strong content validity, high internal consistency (α = 0.828 for sample 1, α = 0.856 for sample 2), and good test-retest reliability (ICC = 0.968, 95% CI: 0.952–0.977). Exploratory factor analysis revealed one component with eigenvalue (3.440) greater than 1, explaining 57.336% of the variance. Confirmatory factor analysis showed good model fit (χ^2^/df = 2.462, RMSEA = 0.054, SRMR = 0.029, CFI = 0.968, TLI = 0.956). The factor loadings of the 6 items ranged from 0.549 to 0.853, all exceeding 0.50, with the lower bounds of their confidence intervals also above 0.50. The SABAS-CV had a strong correlation with the Chinese version of the Nomophobia Questionnaire (r = 0.715) and the SAS-CSV (r = 0.826). Measurement invariance test across gender demonstrated that the SABAS-CV was measurement invariant for male and female college students.

**Conclusion:**

The SABAS-CV serves as a valid instrument for assessing smartphone addiction in Chinese college students, indicating that the SABAS has high applicability in the Chinese cultural context.

## Introduction

Smartphones, as the carrier of mobile Internet development, have become the daily necessities of an increasing number of people in modern society. Smartphones have led to great changes in daily activities and behaviors. Smartphones can fulfill various needs such as entertainment, studying, socializing, and shopping. Nevertheless, overusing smartphones can result in addiction, adversely affecting both physical and mental well-being. Smartphone addiction describes the negative consequences of the lack of control over the use of smartphones [1]. Similar to internet addiction, smartphone addiction is considered as a form of technological or behavioral addiction [1–3]. Smartphone addicts may manifest six core manifestations of addiction: salience, mood modification, tolerance, withdrawal, conflict, and relapse [4,5]. Smartphone addiction refers to the excessive use of a smartphone driven by a strong need (dependence, habituation, and addictive behavior), to the point where an individual is unable to perform essential daily tasks without the constant use of the device, resulting in the neglect of other life areas [1]. Studies have demonstrated that smartphone addiction can impair college students’ concentration in class [6] and reduce their overall well-being [7–9]. In more severe cases, it may lead to social anxiety [10], personality disorders [11], and suicidal ideation [12–14]. Due to the substantial negative effects of smartphone addiction, it is essential to address this issue among college students.

Scholars have created various research instruments to assess smartphone addiction, including the Smartphone Addiction Scale (SAS) and Smartphone Addiction Scale-Short Version (SAS-SV) by Kwon et al. [15,16], the Smartphone Addiction Inventory (SPAI) by Lin et al. [17], the Smartphone Addiction Proneness Scale (SAPS) by Kim et al. [18], and the Smartphone Application-Based Addiction Scale (SABAS) by Csibi et al. [19]. Among these research instruments, the SAS-SV developed by Kwon et al. and the SABAS developed by Csibi et al. are commonly used research instruments for assessing smartphone addiction among college students. The SAS-SV has shown to be effective in measuring smartphone addiction in Turkey [20–23], Egypt [24], Pakistan [25], Britain [26], and China [27,28], while the SABAS has been found to be a valid instrument for measuring smartphone addiction in Malaysia [29,30], Indonesia [31], Bangladesh [32], and China [30,33–36].

Before the SABAS was developed, although scholars developed some instruments to evaluate smartphone addiction, all these instruments had obvious common shortcomings that none of them had any theoretical framework to support their items (they were based on either DSM criteria or prior scales and findings), and the SAS-SV was no exception. A successful addiction theory should (1) integrate pharmacological, cultural, situational, and personality factors, (2) explain the diverse manifestations of addiction across cultures, individuals, and time, (3) address the shared characteristics of all types of addiction, and (4) align with the realities of human experience [5]. Accordingly, Griffiths proposed the “Addiction Component Model”, suggesting that smartphone addiction exhibits the six core features of addiction: salience, mood modification, tolerance, withdrawal, conflict, and relapse [5]. The development of SABAS was based on the “Addiction Component Model”, and each item represented a component of the model [5,19]. Some scholars also argued that individuals are actually addicted to mobile applications (such as social networks and games) rather than to smartphones themselves [19,37]. As claimed by Kuss and Griffiths [37]: “individuals are no more addicted to smartphones than alcoholics are addicted to bottles” (p. 8). Therefore, it seems inappropriate to explain the use of individual smartphones by evaluating the degree of individual addiction to smartphones, and the word “smartphone addiction”, which is widely used by people, cannot be simply understood as addiction to smartphones in the literal sense but as addiction to applications on smartphones. Consequently, compared with the SAS-SV, the SABAS may be a better option for assessing smartphone addiction in college students.

As reported in the 52nd Statistical Report on China’s Internet Development by the China Internet Network Information Center (CINIC), 99.8% of internet users in China utilize smartphones to access the internet [38]. Researches have shown that the prevalence of “smartphone addiction” among Chinese college students is also notably high, ranging from 26.92% to 49.8% [39–44]. Although previous studies have utilized instruments such as the Mobile Phone Addiction Index Scale (MPAIS) to assess smartphone addiction, these scales were originally developed for non-smartphones and do not adequately account for the similarities between smartphones and computers. The Smartphone Addiction Based on Application Scale (SABAS) has been identified as a valid scale for assessing the smartphone addiction of college students in mainland China [30]. However, this study have primarily focused on testing the scale’s reliability and did not scientifically translate them through forward-backward method, much less conduct a cross-cultural research or examine the cross-gender measurement invariance of the scale [30]. Consequently, there is currently no valid Chinese version of smartphone addiction scale specifically designed for Chinese college students. Therefore, it is critical to translate the SABAS into Chinese and evaluate the psychometric properties of the Chinese version, the Smartphone Application-Based Addiction Scale (SABAS-CV), to effectively investigate smartphone addiction among this population.

This study aimed to translate the SABAS into Chinese and examine the psychometric characteristics and measurement invariance across gender of the SABAS-CV in Chinese college students. This will provide a valid instrument for further investigations into smartphone addiction among this population.

## Methods

### Linguistic validation

Using forward-backward translation method, this study translated the SABAS into Chinese [45]. The translation process involved several stages: forward translation, discussion with the translator, backward translation, expert review, and cognitive testing with a carefully selected sample. Initially, the SABAS was translated into Chinese by a native Chinese speaker with expertise in English applied linguistics and professional translation qualifications. This translated version was then reviewed and refined in collaboration with the researcher to produce the preliminary Chinese version. Subsequently, the preliminary Chinese version was translated back into English by another native Chinese speaker, also specialized in English applied linguistics and qualified in translation. The researcher then consulted with two experts in smartphone addiction to review the translations and resolve any discrepancies, resulting in the final Chinese version of the SABAS (SABAS-CV) (S1 File).

The researcher selected 45 college students from a class at Shandong Women’s University as participants for the item review of SABAS-CV through convenient sampling. During the period from March 27th, 2023 to March 30th, 2023, the researcher interviewed the participants after they completed the questionnaire. Based on the item review, the 45 participants took about 1 minute to complete the questionnaire, suggesting that the number of items was suitable. All participants stated that the instructions and questions were clear and easily understandable. Hence, the SABAS-CV is appropriate for the subsequent questionnaire survey.

### Sample and procedure

For this research, 18 classes of college students from Shandong Women’s University and Shandong Management University were chosen using cluster random sampling method. The researcher, with the assistance of class counselors, secured permission from all 18 classes to take part in a questionnaire survey conducted through a QQ group. In this study, participants in the classes were provided with online questionnaires, along with instructions and consent forms, through QQ groups. The students were notified that the questionnaires would be available for completion for a period of two weeks, from April 16, 2023, to April 29, 2023. Finally, 644 valid online questionnaires were gathered from participants aged 18–24 (M = 20.32; SD = 1.432). The entire sample was randomly divided into two groups: sample 1 (n = 322) and sample 2 (n = 322).These groups were employed to evaluate the reliability and validity of the SABAS-CV. Sample 1 was utilized for internal consistency reliability analysis and exploratory factor analysis. Sample 2 was utilized for internal consistency reliability analysis, confirmatory factor analysis, convergent validity analysis, and cross-gender measurement invariance test. To evaluate the test-retest reliability of the SABAS-CV, 80 college students were randomly selected from a pool of 644 Chinese college students. The second measurement was conducted one month after the first. As this study used cluster random sampling method for sampling and the entire sample was randomly divided into two groups for subsequent data analysis, the influence of demographic characteristics on the results of the study could be minimized. The characteristics of the participants in the sample are detailed in Table 1.

**Table 1:** Descriptive statistics of participants (n = 644).

Variable	Levels	n	Percent (%)
Gender	Male	283	43.94
	Female	361	56.06
Grade	Freshman	181	28.11
	Sophomore	170	26.40
	Junior	160	24.84
	Senior	133	20.65
Major	Engineering	205	31.83
	Science	201	31.21
	Liberal arts	238	36.96
Only child/Non-only child	Only child	265	41.15
	Non-only child	379	58.85
Urban/Rural area	Urban area	286	44.41
	Rural area	358	55.59
Household income per month (RMB)	Below 3,000	79	12.27
	3,001–6,000	228	35.40
	6,001–9,000	172	26.71
	9,001–12,000	95	14.75
	Above 12,000	70	10.87

### Instruments

#### Smartphone Application-Based Addiction Scale (SABAS).

The SABAS is a six-item scale designed to evaluate the risk of smartphone addiction, grounded in the components model of addiction [19]. Items were rated on a 6-point Likert scale from 1 (strongly disagree) to 6 (strongly agree). The SABAS was translated into Chinese using the forward-backward method, as described in the linguistic validation section.

#### Chinese version of nomophobia questionnaire.

The nomophobia behaviour of Chinese college students was assessed using the 20-item Chinese Version of Nomophobia Questionnaire [46]. The scale consists of four subscales: not being able to communicate, losing connectedness, not being able to access information, and giving up convenience. In this study, the Cronbach’s alpha coefficient for the overall scale was 0.976. The Cronbach’s alpha coefficients for the four subscales were 0.956, 0.946, 0.925, and 0.943, respectively, and the total scale showed good construct validity: χ^2^/df = 2.17, RMSEA = 0.045 (90% CI: 0.035–0.051), TLI = 0.969, CFI = 0.980, SRMR = 0.036.

#### Smartphone Addiction Scale-Chinese Short Version (SAS-CSV).

In this study, Chinese college students’ smartphone addiction was evaluated using the 10-item SAS-CSV [47]. The scale contains three subscales: tolerance, withdrawal, and negative effect. The Cronbach’s alpha coefficient for the total scale was 0.922, and the alpha coefficients for the tolerance, withdrawal, and negative effect subscales were 0.822, 0.867, and 0.811, respectively. The total scale showed good construct validity: χ^2^/df = 2.36, RMSEA = 0.049 (90% CI: 0.026–0.055), TLI = 0.958, CFI = 0.976, SRMR = 0.039.

### Data analysis

In the present study, data analysis consisted of the following eight sections

#### Content validity.

The content validity index (CVI) was utilized to evaluate the CVI of the SABAS-CV. A panel of four experts, consisting of two developmental and educational psychologists, one psychometrician, and one psychostatistician, was invited by the researcher to assess the content validity of the SABAS-CV. The acceptable level of content validity for items was determined to be a CVI value of 0.75 or higher [48].

#### Descriptive statistics.

This study conducted descriptive statistics analysis of the six SABAS-CV items on sample 1 and sample 2. The descriptive statistical results included mean, standard deviation, skewness, and kurtosis. Among them, the skewness and kurtosis were used to test the normality of sample 1 and sample 2 scores on the six SABAS-CV items. When the absolute value of the skewness and kurtosis of the data for each item is less than 2, it indicates that the data is approaching a normal distribution [49].

#### Floor and ceiling effects.

Ceiling effects were assessed by the percentage of participants scoring in the upper decile, while floor effects were assessed by the percentage of participants scoring in the lower decile. Floor and ceiling effects are considered significant if more than 15% of participants obtain the lowest or highest possible score [50]. A percentage up to 25% is regarded as a moderate effect, while scores exceeding 25% are considered indicative of a severe floor or ceiling effect [51].

#### Internal consistency reliability.

Using the Cronbach’s-α coefficient to assess the internal consistency reliability of the SABAS-CV. A Cronbach’s-α value greater than 0.70 is considered indicative of acceptable reliability for the scale [52].

#### Test-retest reliability.

A follow-up test was performed one month after the initial test. The intraclass correlation coefficient (ICC) suggests that a reliability standard of at least 0.70 is recommended [53].

#### Structural validity.

To assess the SABAS-CV’s structural validity, both EFA and CFA were performed.EFA and CFA were conducted to evaluate the structural validity of the scale. Kaiser-Meyer-Olkin (KMO) test, Bartlett’s spherical test, and EFA were performed using SPSS software (Version 29.0) with the factor extraction method set to “Maximum Likelihood” and the factor rotation method set to “Direct Oblimin”. CFA was performed with Mplus software (8.1). Various fit indices including χ^2^, degree of freedom (df), χ^2^/df, tucker-lewis index (TLI), comparative fit index (CFI), root-mean-square error of approximation (RMSEA), and standardized root mean square residual (SRMR) were computed to assess the model fit. Data suitability for EFA was determined by a Kaiser-Meyer-Olkin (KMO) value exceeding 0.5 and a statistically significant Bartlett’s test of sphericity (p < 0.05) [52]. A good model fit is indicated by χ^2^/df < 3, RMSEA below 0.06, SRMR below 0.05, and CFI and TLI values above 0.95 [54,55].

#### Convergent validity.

The convergent validity of the SABAS-CV was assessed by bootstrap tests using Mplus software (8.1) and calculating the correlation between the total score of the Chinese version of the Nomophobia Questionnaire, the total score of the SAS-CSV, and the total score of the SABAS-CV. When the factor loadings of all 6 items are higher than 0.50 and the lower bounds of the confidence intervals for these factor loadings are also above 0.50, it can be concluded that the scale has good convergent validity [52]. Furthermore, when the correlation coefficient is large and significant, it indicates that the SABAS-CV has good convergent validity [52].

#### Measurement invariance across gender.

First, this study conducted descriptive statistics analysis of the six items of the SABAS-CV in terms of gender to test the normality of scores on the six items of the SABAS-CV for male and female college students. If the absolute values of the skewness and kurtosis of the data are both less than 2, it suggests that the data is nearing a normal distribution. Then, the measurement invariance across gender of the SABAS-CV was tested. Specifically, this study evaluated measurement invariance in four stages: (a) fitting the measurement model independently for each group, (b) testing model 1 for configural invariance, (c) assessing model 2 for metric invariance (equal loadings across gender), and (d) evaluating model 3 for scalar invariance (equal loadings and intercepts across gender). Model fit was determined using the chi-square statistic, TLI, CFI, and RMSEA, with acceptable fit criteria being CFI and TLI above 0.90, SRMR below 0.05, and RMSEA under 0.08 [56]. Measurement invariance was confirmed when the chi-square differences (p > 0.05) and model fit differences (∆CFI < 0.01) were observed between models [52,57,58].

### Ethical consideration

The study was approved by the Research Ethics Committee of School of Education of Shandong Women’s University (ethics approval number: SDWU/SE/EC/20221220). All procedures followed were in accordance with the ethical standards of the responsible committee on human experimentation (institutional and national) and with the Helsinki Declaration of 1975, as revised in 2000 (5). All the participants in this study signed the online informed consent form.

## Results

### Content validity and descriptive statistics

The results of content validity analysis showed that the CVI values of six items of SABAS-CV were all 1, indicating strong content validity for the SABAS-CV.

Descriptive statistical results indicated that the mean (standard deviation) of six items of SABAS-CV ranges from 2.07 to 4.11(1.182 to 1.415) on sample 1. On sample 2, the mean (standard deviation) of the six items of SABAS-CV ranged from 2.44 to 4.20 (1.349 to 1.492). On sample 1, the absolute value of skewness (kurtosis) ranged from 0.103 to 0.949 (0.134 to 0.797), both of which were less than 2. On sample 2, the absolute value of skewness (kurtosis) ranged from 0.063 to 0.977 (0.267 to 0.559), both of which were less than 2. As shown in Table 2, the above results showed that the scores of sample 1 and sample 2 on six items of SABAS-CV were close to normal distribution.

**Table 2:** Descriptive statistics of the item of the SABAS-CV on sample 1 and sample 2.

Item	Mean (Standard Deviation)		Skewness		Kurtosis	
	Sample 1	Sample 2	Sample 1	Sample 2	Sample 1	Sample 2
1	4.11 (1.328)	4.20 (1.372)	−0.480	−0.977	−0.450	0.512
2	2.07 (1.182)	2.44 (1.356)	0.949	0.570	0.134	−0.369
3	3.68 (1.415)	3.77 (1.492)	−0.273	−0.621	−0.797	−0.267
4	3.33 (1.331)	3.45 (1.374)	−0.103	−0.415	−0.624	−0.369
5	2.81 (1.345)	2.92 (1.349)	0.469	−0.063	−0.486	−0.494
6	3.10 (1.336)	3.14 (1.355)	0.345	−0.096	−0.546	−0.559

### Floor effect, ceiling effect, and reliability

For both samples 1 and 2, the total score of the SABAS-CV showed no evidence of floor or ceiling effects. Specifically, as shown in Table 3, for sample 1, the floor effect was 7.8%, and the ceiling effect was 10.9%, both less than 15%. For sample 2, the floor effect was 5.7%, and the ceiling effect was 8.6%, both less than 15%. In conclusion, the SABAS-CV had good discrimination.

**Table 3:** Mean and standard deviation, Cronbach’s α coefficient, and floor/ceiling effect of the SABAS-CV on sample 1 and sample 2.

Mean (Standard Deviation)		Floor/ceiling effect (%)		Cronbach’α	
Sample 1	Sample 2	Sample 1	Sample 2	Sample 1	Sample 2
20.365 (6.291)	20.062 (6.116)	7.8/10.9	5.7/8.6	0.828	0.856

As shown in Table 3, the Cronbach’s α coefficients of SABAS-CV were above 0.70 for both sample 1 and sample 2, demonstrating strong internal consistency. Specifically, the coefficient was 0.828 for sample 1 and 0.856 for sample 2.

The test-retest reliability of SABAS-CV was evaluated using a sample of eighty college students. The results revealed an ICC of 0.968 (95% CI: 0.952–0.977; P < 0.001), indicating that SABAS-CV demonstrated excellent test-retest reliability.

### Structural validity and convergent validity

The results of Bartlett sphere test (KMO = 0.861; χ^2^ = 1653.342; P < 0.001) indicated that the data was very suitable for factor analysis. Through EFA, a component exhibiting an eigenvalue (3.440) was obtained, with the cumulative variance explained reaching 57.336%. The results of CFA showed that χ^2^/df, RMSEA, SRMR, CFI, and TLI all satisfied the criteria for good model fit (χ^2^/df = 2.462, RMSEA = 0.054 (90% CI: 0.032–0.059), SRMR = 0.029, TLI = 0.956). The above results revealed that the SABAS-CV is a unidimensional scale with strong structural validity. Table 4 presents the result of EFA of the SABAS-CV. Table 5 presents the fit indices of the SABAS-CV’s structural equation model.

**Table 4:** EFA of the SABAS-CV (n = 322).

Item	Communality	Factor loading
4.Over time, I fiddle around more and more with my smartphone. 随着时间的推移, 我越来越多地摆弄我的智能手机。	0.756	0.869
5.If I cannot use or access my smartphone when I feel like, I feel sad, moody, or irritable. 如果我不能随心所欲地使用或访问我的智能手机, 我会感到难过、情绪化或易怒。	0.717	0.847
6.If I try to cut the time I use my smartphone, I manage to do so for a while, but then I end up using it as much or more than before. 如果我试图减少使用智能手机的时间, 我会暂时做到这一点, 但随后我会像以前一样频繁甚至更多地使用它。	0.711	0.843
3.Preoccupying myself with my smartphone is a way of changing my mood (I get a buzz, or I can escape or get away, if I need to). 让自己沉浸于智能手机中是我改变心情的一种方式（我可以获得快乐, 或者我可以逃避, 如果有需要的话）。	0.620	0.787
1.My smartphone is the most important thing in my Life. 我的智能手机是我生活中最重要的东西。	0.455	0.596
2.Conflicts have arisen between me and my family (or friends) because of my smartphone use. 因为我的智能手机使用, 我和我的家人（或朋友）之间出现了冲突。	0.382	0.531
Eigenvalue		3.440
Variance contribution (%)		57.336

**Table 5:** Fit indices of the SABAS-CV’s structural equation model (n = 322).

Fitness index	χ^2^	df	p	χ^2^/df	*CFI*	*TLI*	*RMSEA* (95% CI)	*SRMR*
Criteria			< 0.05	< 3	> 0.95	> 0.95	< 0.06	< 0.05
Model	22.157	9	0.000	2.462	0.968	0.956	0.054 (0.032-0.059)	0.029

As shown in Table 6, the factor loadings of the 6 items ranged from 0.549 to 0.853, all of which were higher than 0.50. Additionally, the lower bounds of the confidence intervals for the factor loadings of these items were also above 0.50. In addition, the correlation analysis results (see Table 7) indicated that the correlation coefficient between the SABAS-CV and the Chinese Version of the Nomophobia Questionnaire was 0.715, and the correlation coefficient between the SABAS-CV and the SAS-CSV was 0.826. These results demonstrated that the SABAS-CV exhibited good convergent validity.

**Table 6:** The factor loadings of the six items and their corresponding confidence intervals.

Item	Factor loadings	Lower	Upper
Item 1	0.571	0.538	0.773
Item 2	0.549	0.520	0.733
Item 3	0.761	0.666	0.835
Item 4	0.853	0.789	0.902
Item 5	0.770	0.671	0.841
Item 6	0.803	0.725	0.861

**Table 7:** Convergent validity of the SABAS-CV.

Scale	1	2	3
1. SABAS-CV	1		
2. NQ-CV	0.715**	1	
3. SAS-CSV	0.826**	0.780**	1

**Note:** SABAS-CV = Chinese Version of Smartphone Application-Based Addiction Scale, NQ-CV = Chinese Version of Nomophobia Questionnaire, SAS-CSV = Smartphone Addiction Scale-Chinese Short Version.

** *p *< 0.01.

### Measurement invariance across gender

Table 8 presents the descriptive statistics of the item of SABAS-CV on gender. Specifically, the mean (standard deviation) of the six items of SABAS-CV among male college students ranged from 2.08 to 3.82 (1.201 to 1.784). The mean (standard deviation) of the six items of SABAS-CV among female college students ranged from 2.39 to 4.42 (1.313 to 1.457). Among male college students, the absolute value of skewness (kurtosis) ranged from 0.252 to 0.792 (0.526 to 1.311), both of which were less than 2. Furthermore, among female college students, the absolute value of skewness (kurtosis) ranged from 0.108 to 0.751 (0.056 to 0.647), both of which were less than 2. These results suggested that the scores of the six items of SABAS-CV in both male and female college students were close to normal distribution.

**Table 8:** Descriptive statistics of the item of SABAS-CV on gender.

Item	Mean (Standard Deviation)		Skewness		Kurtosis	
	Male	Female	Male	Female	Male	Female
1	3.82 (1.586)	4.42 (1.337)	−0.341	−0.719	−0.904	−0.056
2	2.08 (1.201)	2.39 (1.313)	0.792	0.751	−0.526	−0.151
3	3.57 (1.784)	3.85 (1.457)	−0.252	−0.442	−1.311	−0.531
4	3.07 (1.569)	3.64 (1.369)	0.343	−0.267	−0.774	−0.500
5	2.57 (1.658)	3.10 (1.382)	0.672	0.142	−0.735	−0.640
6	3.02 (1.668)	3.20 (1.367)	0.263	0.108	−1.087	−0.647

The measurement model demonstrated a good fit to the data for both male and female college students (see Table 9). For male college students, the model fit is as follows: χ^2^(df = 9) = 18.120, p < 0.001, CFI = 0.953, TLI = 0.952, RMSEA = 0.051, SRMR = 0.049; for female college students, the model fit is as follows: χ^2^(df = 9) = 23.904, p < 0.001, CFI = 0.962, TLI = 0.956, RMSEA = 0.050, SRMR = 0.031. Consequently, the measurement equivalence test can be carried out next.

**Table 9:** Measurement invariance test across gender.

Model tested	Model fit measures							Model differences			
	χ^2^	df	p	CFI	TLI	RMSEA	SRMR	△CFI	△χ^2^	△df	p
Separate groups											
Male	18.120	9	0.000	0.953	0.952	0.051	0.049				
Female	23.904	9	0.000	0.962	0.956	0.050	0.031				
Model 1: Configural invariance	42.024	18	0.000	0.961	0.955	0.053	0.033				
Model 2: Metric invariance	45.130	23	0.000	0.962	0.950	0.051	0.038	< 0.01	3.106	5	0.684
Model 3: Scalar invariance	55.007	28	0.000	0.958	0.955	0.059	0.044	< 0.01	9.877	5	0.079

Models 1, 2, and 3 all demonstrated good fit. A comparison between Models 2 and 1 revealed no significant differences in fit indices (∆χ^2^ = 3.106, ∆df = 5, p > 0.05, ∆CFI < 0.01). Similarly, comparing Models 3 and 2 showed no significant differences in fit indices (∆χ2 = 9.877, ∆df = 5, p > 0.05, ∆CFI < 0.01). These findings confirm that the SABAS-CV achieves scalar invariance (strong invariance) across gender.

## Discussion

In this study, the SABAS was first translated into Chinese, and then the content validity of the six items in the SABAS-CV was evaluated by the panel of experts. The evaluation revealed that all six items of the SABAS-CV had a CVI value of 1, indicating strong content validity. The SABAS-CV demonstrated strong content validity, likely due to its foundation on the “Addiction Component Model” in the original study [19]. Each item in the scale corresponds to a component of the “Addiction Component Model” [5]. Regarding the reliability of the SABAS-CV, the results indicated that the Cronbach’s-α coefficients of the SABAS-CV in sample 1 and sample 2 were 0.828 and 0.856, respectively. These values exceeded the threshold of 0.70 and were consistent with previous cross-cultural research on the SABAS in Italy, Indonesia, and Serbia. Specifically, the Cronbach’s-α coefficient for the Italian version of SABAS was 0.89 [59], for the Indonesian version it was 0.74 [31], and for the Serbian version it was 0.81 [60]. The intraclass correlation coefficient (ICC) of the SABAS-CV was 0.968 (95% CI: 0.952–0.977; P < 0.001), demonstrating strong test-retest reliability for the SABAS-CV. The findings in Serbia were partially supported by this result (ICC: 0.795; 95% CI: 0.731–0.844) [60]. Despite the ICC value being higher in this study compared to Serbia, both studies confirmed that the SABAS demonstrated good test-retest reliability. These consistent results support the applicability of the SABAS-CV in various settings, enabling reliable assessment of smartphone addiction among Chinese populations and facilitating cross-cultural comparisons. The findings highlight the utility of this scale in research, clinical practice, and policy-making, particularly in designing interventions and understanding the global prevalence of smartphone addiction. The high reliability of the SABAS-CV may be attributed to the researchers’ adherence to the forward-backward translation process, which guaranteed translation correctness.

With regard to the structural validity of the SABAS-CV, the orginal study did not conduct confirmatory factor analysis [19]. Csibi et al. only evaluated the internal consistency reliability of the SABAS and examined the structural validity of the SABAS through exploratory factor analyses in the original study [19]. This study indicated that the SABAS-CV is a unidimensional scale, as confirmed by the results of the original study and cross-cultural research conducted in Italy, Indonesia, and Serbia. [31,59,60]. The six items of the SABAS-CV explained 57.336% of variance variation. The fit indices of the SABAS-CV’s structural equation model all satisfied the standard of good model fit: CFI (0.968) and TLI (0.956) exceeded 0.95, RMSEA (0.054; 95%CI: 0.032–0.059) was below 0.06, and SRMR (0.029) was under 0.05. These results, combined with previous cross-cultural studies in Italy, Indonesia, and Serbia, confirmed the strong structural validity of the SABAS. The strong structural validity of the SABAS-CV can be credited to the use of a representative sample obtained through cluster random sampling. This sample was then randomly divided into two samples for EFA and CFA, ultimately ensuring the good structural validity of the SABAS-CV in the study.

In this study, the convergent validity analysis also verified the good validity of the SABAS-CV. Specifically, the factor loadings of the 6 items ranged from 0.549 to 0.853, all exceeding the threshold of 0.50, with the lower bounds of their confidence intervals also above 0.50. This suggests that the items effectively measure the intended construct, and the scale is reliable for assessing the target phenomenon. Besides, the correlation coefficient between the total score of the SABAS-CV and the total score of the Chinese version of the Nomophobia Questionnaire was 0.715, while the correlation coefficient between the total score of the SABAS-CV and the total score of the SAS-CSV was 0.826. These findings are consistent with previous cross-cultural studies that have assessed the convergent validity of the SABAS. For instance, Soraci et al. evaluated the convergent validity of the Serbian version of the SABAS by conducting a Pearson correlation analysis on the total scores of the SABAS-CV and smartphone use [60]. In a similar approach, Nurmala et al. assessed the convergent validity of the Indonesian version of the SABAS by calculating the Pearson correlation coefficient between the total scores of the Depression, Anxiety, Stress Scale, the Nomophobia Questionnaire, and the SABAS-CV [31]. This consistency highlights the SABAS-CV’s stable validity and cross-cultural applicability, supporting its use in assessing smartphone addiction and related constructs in diverse populations. In summary, this scale has demonstrated strong convergent validity across various cultural contexts.

This study reveals that the one-dimensional structure of the SABAS-CV was measurement invariant for male and female college students. Although female college students scored higher than male college students on each item, this gender difference merely reflects gender differences in the severity of smartphone addiction, but it is not measurement bias. Previous research has indicated that male and female smartphone users have different content preferences, with males preferring gaming apps and females preferring social media apps, which may account for these gender differences [61]. Therefore, the SABAS-CV can be used to measure and compare the extent of smartphone addiction across genders in a sample of Chinese college students. The current results support the use of the SABAS-CV in research and clinical settings because it is free of measurement bias in measuring smartphone addiction among male and female college students.

In summary, this study expands existing knowledge in the field of smartphone addiction and offers new perspectives by validating the SABAS cross-culturally among Chinese college students. Firstly, the development of the SABAS is based on Griffiths’ “Addiction Component Model,” which breaks down smartphone addiction into six core features (salience, mood modification, tolerance, withdrawal, conflict, and relapse). This theoretical foundation addresses the lack of theoretical support in previous scales, providing a more scientific and systematic framework for assessing smartphone addiction. Secondly, the study challenges the superficial understanding of “smartphone addiction,” suggesting that individuals may not be addicted to the smartphone itself but rather to its applications (e.g., social networks). This perspective is validated by the SABAS’s strong applicability among Chinese college students. Thus, as an assessment instrument grounded in the “Addiction Component Model,” the SABAS can more accurately capture individuals’ dependency behaviors related to smartphone use, making it potentially more suitable than scales like the SAS-SV for evaluating smartphone addiction among college students. Finally, the study validates the reliability and validity of the SABAS in Chinese college students, demonstrating its high applicability within the Chinese cultural context. This supports the cross-cultural universality of the “Addiction Component Model” and provides a reliable instrument and theoretical basis for future research in China and other cultural settings. In summary, this study contributes new perspectives and methodological support to smartphone addiction research through cross-cultural validation.

### Limitations and future directions

There are several limitations in this study that should be acknowledged. Firstly, this study is based on data from only two universities in Jinan, Shandong Province, China, and the sample may be limited in terms of geographic scope, types of universities, student backgrounds, and cultural characteristics, and may not adequately reflect the situation of students in other regions of China or in a wider range of universities. Future studies should focus on including a more diverse sample to validate the findings of this research. Secondly, the study focused solely on college students, but smartphone addiction is a concern across various age groups (children, adolescents, and adults). Therefore, it is important to assess the cross-gender invariance of the SABAS-CV in different populations. Thirdly, the absence of longitudinal validation limits the ability to determine the stability of the SABAS-CV over time. Future research should employ longitudinal methods to further examine whether the SABAS-CV maintains good reliability and validity consistently. Fourthly, there is a lack of qualitative analysis that could enrich the quantitative research findings. Future studies can adopt qualitative research methods (e.g., focus group interview) to further endorse the quantitative results of this study. Fifthly, this study was only a cross-cultural study of the SABAS in the context of Chinese culture, and did not further investigate the causal relationships and predictors of smartphone addiction, nor did it conduct an intervention study based on the SABAS-CV. Future studies can fully utilize this scale to explore research questions related to smartphone addiction.

## Conclusion

The Smartphone Application-Based Addiction Scale (SABAS) was developed based on Griffiths’ “Addiction Component Model”, addressing the lack of theoretical foundation in previous scales. As a more theoretically grounded and culturally adaptive assessment instrument, the SABAS is better suited for evaluating smartphone addiction among college students. This study validated the Chinese version of the Smartphone Application-Based Addiction Scale (SABAS-CV) in a sample of Chinese college students. The results demonstrated that the SABAS-CV exhibited strong reliability and validity, as well as measurement invariance across gender. Therefore, it can be considered an effective instrument for assessing smartphone addiction among Chinese college students. This study not only validated the “Addiction Component Model” within the Chinese cultural context but also provided support for the perspective that “individuals are actually addicted to mobile applications rather than the smartphone itself”. Furthermore, from a public health perspective, addressing smartphone addiction among college students is of critical importance. The SABAS-CV can serve as a powerful tool for developing educational interventions and public health policies. Future research should further explore the underlying causes of smartphone addiction and develop targeted intervention strategies to address this increasingly prominent public health issue.

## Supporting information

S1 File Chinese version of Smartphone Application-Based Addiction Scale. (DOCX)
