# Supplementary material for: Psychometric properties and measurement invariance across gender of the Chinese version of the Smartphone Application-Based Addiction Scale (SABAS) among Chinese college students
Source: PLoS One. 2025 May 21;20(5):e0323215. doi: 10.1371/journal.pone.0323215 (PMC12094724; doi:10.1371/journal.pone.0323215)
Supplement: S1 File — (DOCX) [file pone.0323215.s001.docx]

**中文版智能手机应用成瘾量表**

为了调查大学生智能手机成瘾的状况，现需要您填写下面的调查问卷。以下问卷中的问题是您熟悉的内容，一共有5个选项: 1为完全不符合；2为有点符合；3为比较符合；4为符合；5为完全符合，请勾选相应的选项。

|  | 完全  不符合 | 有点  符合 | 比较  符合 | 符合 | 完全  符合 |
| --- | --- | --- | --- | --- | --- |
|  | 1 | 2 | 3 | 4 | 5 |
| 1. 我的智能手机是我生活中最重要的东西。 |  |  |  |  |  |
| 1. 因为我的智能手机使用，我和我的家人（或朋友）之间出现了冲突。 |  |  |  |  |  |
| 1. 让自己沉浸于智能手机中是我改变心情的一种方式（我可以获得快乐，或者我可以逃避，如果有需要的话）。 |  |  |  |  |  |
| 1. 随着时间的推移，我越来越多地摆弄我的智能手机。 |  |  |  |  |  |
| 1. 如果我不能随心所欲地使用或访问我的智能手机，我会感到难过、情绪化或易怒。 |  |  |  |  |  |
| 1. 如果我试图减少使用智能手机的时间，我会暂时做到这一点，但随后我会像以前一样频繁甚至更多地使用它。 |  |  |  |  |  |
